# Alleviating Effect of *Lactiplantibacillus plantarum* HYY-S10 on Colitis in Mice Based on an Analysis of the Immune Axis in the Intestine

**DOI:** 10.3390/microorganisms13040840

**Published:** 2025-04-07

**Authors:** Mengna Li, Xintong Liu, Weijian Chen, Haoyue Xu, Fang Huang, Qingbo Yao, Xiangze Jia, Yanyan Huang

**Affiliations:** 1 Guangdong Provincial Key Laboratory of Intelligent Food Manufacturing, College of Food Science and Engineering, Foshan University, Foshan 528225, China; mengnlimo@126.com (M.L.);; 2 School of Food Sciences and Engineering, South China University of Technology, Guangzhou 510640, China

**Keywords:** Yunnan De’ang Sour Tea, colitis, *Lactiplantibacillus plantarum* HYY-S10, probiotics, intestinal flora

## Abstract

The pathogenesis of ulcerative colitis (UC) has been fundamentally associated with intestinal microbiota dysbiosis and disruption of immune homeostasis. This study systematically investigates the therapeutic potential of *Lactiplantibacillus plantarum* HYY-S10 (HYY-S10), a novel strain isolated from De’ang sour tea in Yun an, China, with a focus on its mechanisms for alleviating colitis through the modulation of gut microbiota. Using a dextran sulfate sodium (DSS)-induced colitis model in C57BL/6J mice, our findings demonstrated that seven days of oral supplementation with HYY-S10 (1 × 10^8^ CFU/mL, 0.2 mL/10 g body weight) significantly improved Disease Activity Index (DAI) scores and attenuated characteristic colitis symptoms, including progressive weight loss, rectal bleeding, and abnormal stool consistency. Administration of HYY-S10 exhibited significant immunomodulatory effects characterized by the downregulation of pro-inflammatory mediators (such as IL-1β, IL-6, IFN-γ, and LPS) while concomitantly upregulating anti-inflammatory IL-10 expression. Additionally, the strain enhanced intestinal antioxidant capacity by increasing GSH-Px activity, which collectively contributed to the reduction in intestinal inflammation. Furthermore, HYY-S10 demonstrated multifaceted protective effects by ameliorating oxidative stress through the restoration of redox homeostasis and modulation of gut microbial ecology. Probiotic intervention significantly increased short-chain fatty acids (SCFAs) production and notably enhanced the relative abundance of beneficial taxa, including *Akkermansia* and *Ruminococcus*_B, while restoring microbial diversity and ecological stability. Collectively, our results demonstrate that HYY-S10 alleviates experimental colitis by modulating the intestinal immune axis and microbiota composition, providing mechanistic insights to support its potential as a probiotic-based therapeutic strategy for UC.

## 1. Introduction

Ulcerative colitis (UC), a chronic immune-mediated disorder of the intestinal tract, is clinically characterized by recurrent mucopurulent bloody diarrhea, tenesmus, and abdominal pain. Pathologically, it is marked by diffuse continuous inflammation that extends proximally from the rectum [1]. The pathogenesis of UC is complex and involves multifactorial interactions among environment factors, genetics predispositions, gut microbiota, and the immune system [2]. With changes in lifestyle and dietary habits, the incidence of UC is rising globally, particularly in developed countries and regions [3]. Recurrent episodes of UC not only significantly reduce the quality of life of patients but may also increase the risk of colorectal cancer lesions; thus, exploring effective treatment strategies is imperative.

Impaired intestinal barrier function represents a fundamental pathological feature of UC, characterized by various disruptions across mechanical, chemical, immune, and biological barriers [4]. Among these components, the intestinal immune barrier is crucial for maintaining gut homeostasis and defending against pathogen invasion [5]. The intestinal immune system regulates the balance between immune tolerance and inflammatory response through complex cellular and molecular mechanisms [6]. In patients with UC, dysregulation of the gut–immune axis leads to abnormal functioning of immune cells such as macrophages, dendritic cells, and T cells, subsequently triggering an excessive inflammatory response [7]. Furthermore, the compromise of gut barrier function further exacerbates the abnormal activation of the immune system, thereby creating a vicious cycle. Therefore, restoring immune homeostasis and repairing intestinal barrier function by modulating the gut–immune axis has become an important line of research for the treatment of UC.

Recently, increasing research has focused on the interaction between the gut microbiota and the immune system, leading to a gradual elucidation of the mechanisms by which probiotics alleviate UC. Studies show that probiotics exert significant effects in relieving UC through various pathways, including the regulation of intestinal flora balance, enhancement of intestinal barrier function, and modulation of immune responses [8]. Among these probiotics, *Lactiplantibacillus plantarum*, commonly found in food and human environments, has shown effectiveness in ameliorating symptoms of UC and promoting recovery of intestinal barrier function [9]. The therapeutic mechanisms of *Lactiplantibacillus plantarum* encompass multiple biological pathways, including immunomodulatory effects through cytokine regulation, attenuation of reactive oxygen species-mediated damage, enhancement of intestinal barrier integrity via upregulation of tight-junction proteins, stimulation of goblet-cell-derived mucin secretion, modulation of gut microbial composition, and biosynthesis of short-chain fatty acids (SCFAs) [10]. A study conducted by Min et al. (2023) [11] investigated the mechanism of action of *Lactiplantibacillus plantarum* NCU1125 in repairing intestinal barrier damage in mice. The findings revealed that NCU1125 enhanced intestinal epithelial barrier integrity by upregulating the expression of critical tight-junction proteins, including ZO-1, occludin, and claudin-1, and promoting the secretion of the protective mucin Muc2. In addition, NCU1125 was able to further improve intestinal immune barrier function by regulating Th17/Treg cell balance and promoting secretory immunoglobulin A production. These findings provide an experimental basis for the potential use of *Lactiplantibacillus plantarum* in intestinal barrier repair. In addition, a study by Wong et al. (2024) [12] further revealed the multiple mechanisms of action of *Lactiplantibacillus plantarum* HKL-137 in regulating intestinal flora and alleviating colitis. The experimental data demonstrate that administration of HKL-137 markedly enhanced the relative proportions of *Lactobacillus* and *Firmicutes* within the intestinal microbiota, thereby effectively modulating the dysbiosis in murine colitis models. In addition, HKL-137 significantly attenuated the inflammatory response in colitis mice by inhibiting the expression of key pro-inflammatory cytokines, such as IL-6 and TNF-α.

During the preliminary investigation, a novel *Lactiplantibacillus plantarum* HYY-S10 (HYY-S10) with significant probiotic potential was successfully isolated from Yunnan De’ang Sour Tea. The isolate demonstrated exceptional gastrointestinal tolerance in simulated digestive conditions and exhibited superior intestinal colonization capacity. To elucidate the therapeutic mechanisms of HYY-S10 in colitis management and gut microbiota modulation, a DSS-induced murine colitis model was established for subsequent experimental investigations.

## 2. Materials and Methods

### 2.1. HYY-S10 Lyophilized Bacterial Powder Preparation

HYY-S10 (GDMCC 62784) was isolated and screened from Yunnan De’ang sour tea by the school of food science and engineering, Foshan university, and conserved in the Guangdong provincial microbiology conservation center. The strain was inoculated in MRS liquid medium at 2% inoculum (*v*/*v*) and cultured at 37 °C until logarithmic growth. Subsequently, the organisms were collected by centrifugation at 4 °C and 5000× *g* for 15 min for washing. Then, the bacteria were resuspended in lyophilized protective agent and vacuum freeze-dried to prepare HYY-S10 lyophilized bacterial powder, which was stored at −80 °C. A small amount of the powder was reconstituted in sterile water, and the number of viable bacteria in the lyophilized powder was determined to be 1 × 10^8^ CFU/mL by plate counting method.

### 2.2. Animal Experimental Design

**Figure 1:**
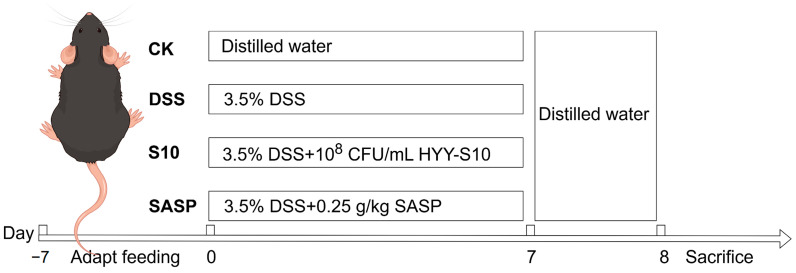
Experimental design table.

C57BL/6J SPF-grade male mice, aged five weeks, were obtained from Guangdong Viton Lihua Laboratory Animal Technology Co., Ltd. (Foshan City, China). The mice were acclimated to a 25 ± 0.5 °C light–dark cycle for seven days upon arrival. Subsequently, the mice were randomly assigned to four groups, each consisting of eight individuals. Both dextran sodium sulfate (DSS, CAS 9011-18-1, MW 40,000; Shanghai Aladdin Biochemical Technology Co., Ltd., Shanghai, China) and sulfasalazine (Shanghai Xinyi Tianping Pharmaceutical Co., Ltd., Shanghai, China) were dissolved in sterile water and filtered through a 0.22 μm membrane for sterilization. HYY-S10 solution was used and was prepared using sterile water to formulate a suspension of 1 × 10^8^ CFU/mL. The experimental protocol is depicted in Figure 1 The CK was orally administered sterile distilled water for seven days to establish a baseline. In contrast, the DSS group received a 3.5% (*w*/*v*) DSS solution orally for seven days to induce colitis. The S10 group was co-administered 0.2 mL/10 g of HYY-S10 (10^8^ CFU/mL) and the 3.5% (*w*/*v*) DSS solution daily for seven days. Meanwhile, the SASP group was treated with 0.25 g/kg sulfasalazine pyridine solution in combination with the DSS solution for the same duration.

### 2.3. Disease Activity Index (DAI) and Sample Collection

During modelling, changes in the mice were regularly recorded using the Yuan et al. [13] DAI scale (Table 1). Mice were anaesthetized with 40 mg/kg sodium pentobarbital (Shanghai Xinyi Tianping Pharmaceutical Co., Ltd.) on day 8 and killed by cervical dislocation. Whole blood samples were collected through retro-orbital bleeding, immediately stored at 4 °C, and centrifuged at 2500× *g* for 10 min to isolate serum. Following euthanasia, a comprehensive necropsy was performed, during which the major organs (kidneys, liver, pancreas, and spleen) were excised and weighed. Intestinal tissue specimens were rapidly frozen in liquid nitrogen and stored at −80 °C to ensure optimal preservation for subsequent biochemical and molecular analyses.

### 2.4. Histopathological Testing

Fresh colonic tissues were collected from 1 cm of the anal side of mice. Tissue samples were immersed in 4% paraformaldehyde fixative at a 1:10 (*w*/*v*) ratio for 24 h at 4 °C, following the method of Gong et al. [14]. After fixation, the colon tissues were embedded in conventional paraffin and sectioned separately at 3 μm using a microtome. The tissue sections were deparaffinized, rehydrated through a graded alcohol series, and H&E stained for histological analysis. The tissue sections were dehydrated, cleared, and mounted with neutral gum. Morphological alterations in colonic tissues were examined under a light microscope.

### 2.5. Measurement of Inflammatory Factors and Oxidative Stress Parameters

Serum concentrations of glutathione peroxidase (GSH-Px), myeloperoxidase (MPO), nitric oxide (NO), lipopolysaccharide (LPS), interleukin IL-10, IL-1β, IL-6, and IFN-γ were quantified using ELISA kits (Shanghai Enzyme-linked Biotechnology Co., Ltd., Shanghai, China). Briefly, 100 μL of standard solutions or serum samples were dispensed into designated wells of a pre-coated microplate, followed by the addition of 100 μL biotin-conjugated detection antibodies to each well. After thorough mixing, the plate was incubated at 37 °C for 60 min. Unbound components were removed by five sequential washes with wash buffer, and residual liquid was aspirated between washes. Subsequently, 100 μL of horseradish peroxidase (HRP)-labeled streptavidin was added to each well and incubated at 37 °C for 30 min. Following another five washing cycles, 100 μL of 3,3′,5,5′-tetramethylbenzidine (TMB) substrate was added, and chromogenic reactions were developed in the dark for 15–30 min. Reactions were terminated by adding 50 μL of 2 M H_2_SO_4_, and absorbance was measured at 450 nm using a microplate reader. All assays included blank controls, and triplicate measurements were performed to calculate mean values.

### 2.6. Determination of SCFAs

SCFAs a group of microbial-derived metabolites, are synthesized through anaerobic fermentation of indigestible carbohydrates, particularly dietary fibers, by commensal gut microbiota. These bioactive compounds play crucial roles in modulating various physiological processes and maintaining host homeostasis. Following a slight modification of the method of Li et al. [15], mouse feces were freeze-dried, resuspended, acidified, extracted and analyzed by high-performance liquid chromatography (1220 Infinity II, Agilent, Santa Clara, CA, USA) on an Organic Acid H+ column (8 μm, 300 × 7.8 mm, Guangzhou Starchrom Technologies Co., Ltd., Guangzhou, China) at a temperature of 55 °C and a flow rate of 0.5 mL/min with 5 mmol/L H_2_SO_4_ as mobile phase for the determination of SCFAs in mouse feces.

### 2.7. 16S rRNA Sequencing

Total genomic DNA was isolated from fecal samples using the Omega DNA Extraction Kit (Omega Bio-tek, Norcross, GA, USA). The V3-V4 hypervariable regions of the bacterial 16S rRNA gene were amplified via polymerase chain reaction (PCR) with universal primers 338F (5′-ACCTACGGGAGGCAGCA-3′) and 806R (5′-GGACTACHVGGGTWTCTAAT-3′). PCR amplification was performed under the following thermal cycling conditions: initial denaturation at 98 °C for 1 min; 30 cycles of denaturation (98 °C for 10 s), annealing (50°C for 30 s), and extension (72 °C for 30 s); and a final extension at 72 °C for 5 min. Amplified products were verified by electrophoresis on a 2% agarose gel (Biowest, Barcelona model, Barcelona, Spain) after mixing with Phusion™ High-Fidelity PCR Master Mix (E7370L, New England Biolabs, Ipswich, MA, USA) in a 1:1 ratio. Equimolar concentrations of PCR amplicons were pooled and purified using the Universal DNA Purification Kit (DP214, TianGen, Beijing, China). Sequencing libraries were prepared using the NEB Next^®^ Ultra DNA Library Prep Kit (Illumina, San Diego, CA, USA). Sequencing service was provided by Wekemo Tech Group Co., Ltd. Shenzhen, China. The data were analyzed on the free online platform of Wekemo Bioincloud (https://www.bioincloud.tech accessed on 10 February 2025).

### 2.8. Statistical Analysis

Data were statistically analyzed (mean ± standard deviation) using SPSS 27.0.1 (SPSS Inc., Chicago, IL, USA), with one-way ANOVA and Tukey’s test between groups (*p* < 0.05). GraphPad Prism 10.2.3 (GraphPad Software, San Diego, CA, USA) and QIIME software (2022.2, University of Colorado Boulder, Boulder, CO, Canada) were used to draw the graphs. Abstract drawing using Figdraw 2.0 (Home for Researchers, Hangzhou, China).

## 3. Results and Discussion

### 3.1. Changes in Body Weight and DAI Score in Mice

**Figure 2:**
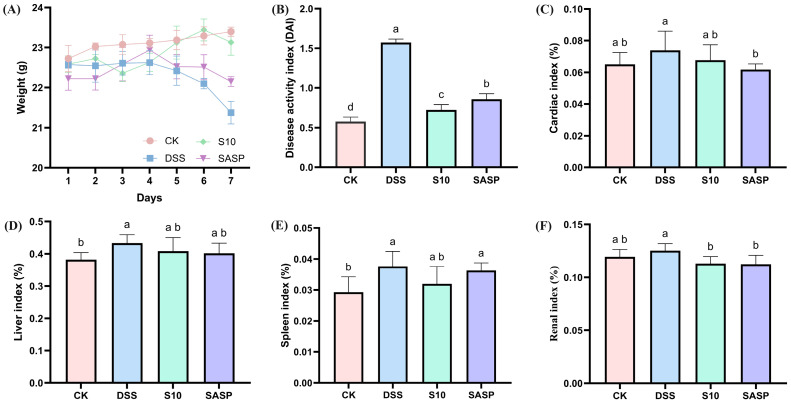
Therapeutic evaluation of *Lactiplantibacillus plantarum* HYY-S10 (HYY-S10) in mice with colitis. (**A**) Rate of change in body weight of mice; (**B**) DAI index; (**C**) Cardiac index; (**D**) Hepatic index; (**E**) Splenic index; and (**F**) Renal index. The letters a,b,c,d indicated significant difference between different groups (*p* < 0.05).

The DAI represents a comprehensive quantitative assessment tool for monitoring colonic inflammation severity in murine models, incorporating three clinically relevant parameters: percentage body weight reduction; stool consistency alterations; and fecal blood [16]. As shown in Figure 2A,B, the DAI scores of mice in the colitis model exhibited a statistically significant increase after seven days of consuming 3.5% DSS drinking water disturbance (*p* < 0.05). Following five days of DSS administration, the model group exhibited characteristic clinical manifestations of colitis, including significant body weight reduction, hematochezia, and decreased fecal output compared to CK group animals. HYY-S10 significantly alleviated DSS-induced body weight loss, reduced fecal blood content, improved stool consistency, and lowered DAI scores compared to the DSS group (*p* < 0.05). Furthermore, HYY-S10 exhibited superior efficacy in alleviating colitis symptoms in mice from days 5 to 7 relative to treatment with sulfasalazine.

### 3.2. Effect of HYY-S10 on Immune Organ Index of Mice with Colitis

Abnormal immune organ indices may indirectly indicate the inflammatory status of the organism. The findings from this study show that the cardiac index (Figure 2C), liver index (Figure 2D), spleen index (Figure 2E), and kidney index (Figure 2F) were significantly elevated in mice subjected to the DSS-induced colitis model, indicating the presence of inflammation in these subjects. Therapeutic intervention with HYY-S10 demonstrated significant anti-colitis efficacy, as evidenced by a marked reduction in immune organ indices compared to DSS (*p* < 0.05).

### 3.3. HYY-S10 Improvement of Colonic Injury in Mice

**Figure 3:**
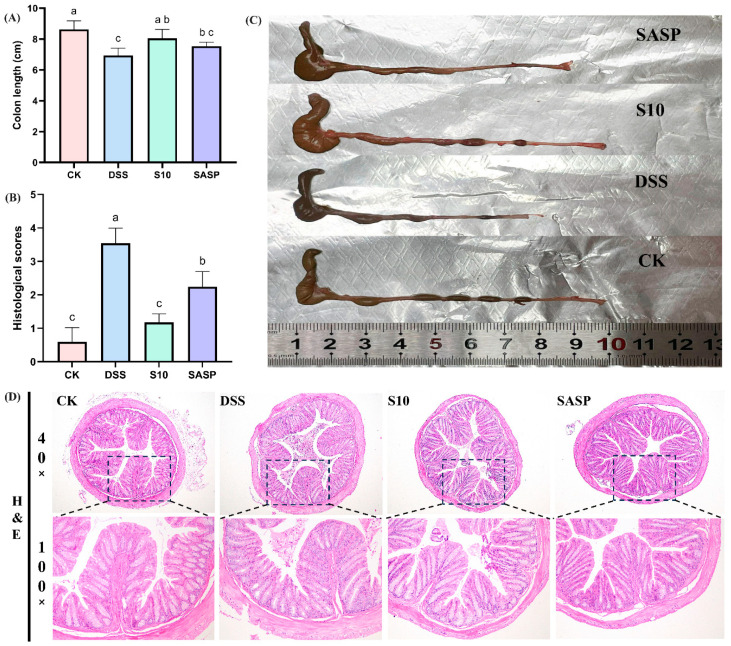
Pathological characteristics of mouse colon. (**A**) Colon length; (**B**) Histological scores; (**C**) Representative view of the colon; (**D**) Color chart of the colon (40× and 100× magnification). The letters a,b,c indicated significant difference between different groups (*p* < 0.05).

The induction of colitis in mice through the administration of DSS results in a notable reduction in colon length, which is a crucial indicator for assessing the severity of colitis [17]. A comparative analysis between the CK and DSS groups of mice revealed a significant decrease in colon length in the latter (Figure 3A). Additionally, the DSS group exhibited dark red, dilute stools within the colon lumen (Figure 3C). The administration of HYY-S10 was observed to significantly alleviate colon shortening caused by DSS. The extent of pathological damage from colitis in mice was evaluated through H&E staining of colon sections (Figure 3D). Mice in the CK group exhibited colons with normal morphology, characterized by intact intestinal epithelium, well-structured crypts, and a high density of goblet cells. No discernible pathological alterations or inflammatory reactions were present. In contrast, the DSS group exhibited intestinal mucosal edema, crypt damage, loss of goblet cells, and significant inflammatory cell infiltration. The histological score for the DSS group improved from 0.6 ± 0.41 to 3.54 ± 0.45 compared to the CK group (0.6 ± 0.41) (Figure 3B). The SASP group effectively preserved colonic histology following sulfasalazine administration. However, some crypt damage, as well as inflammatory cell infiltration, was still observed, resulting in a histological score of 2. In contrast, the administration of HYY-S10 resulted in a notable improvement in intestinal mucosal damage. However, HYY-S10 significantly ameliorated intestinal mucosal damage, as evidenced by the partial restoration of crypt structures and the presence of goblet cells, with the histological score markedly reduced to 1.18 ± 0.25. These results indicate that a dose of 1 × 10^8^ CFU/mL HYY-S10 effectively alleviated pathological damage in mice with colitis.

### 3.4. HYY-S10 Improves Oxidative Stress in Mouse Intestine

**Figure 4:**
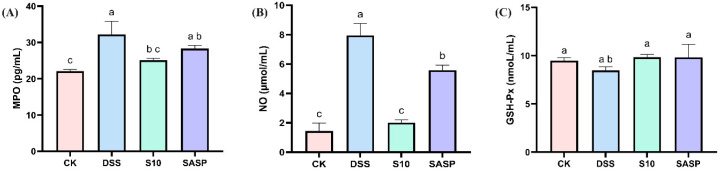
Effect of *Lactiplantibacillus plantarum* HYY-S10 on antioxidant factors in serum of colitis mice. (**A**) MPO; (**B**) NO; (**C**) GSH-Px. The letters a,b,c indicated significant difference between different groups (*p* < 0.05).

In this study, DSS was found to disrupt redox reactions in the organism, resulting in a significant increase in MPO activity and NO levels, accompanied by a decrease in GSH-Px activity. These findings were further corroborated by H&E staining of colon tissue. In addition, oxidative stress promotes the growth and proliferation of aerobic microorganisms (specifically *Proteobacteria*) in the gut flora. Treatment with HYY-S10 intervention significantly restored the equilibrium of MPO (Figure 4A), GSH-Px (Figure 4B), and NO (Figure 4C) activity levels compared to the DSS group. HYY-S10 alleviates disease severity in colitis mice by improving oxidative stress levels.

### 3.5. HYY-S10 Improves Intestinal Inflammatory Response in Mice

**Figure 5:**
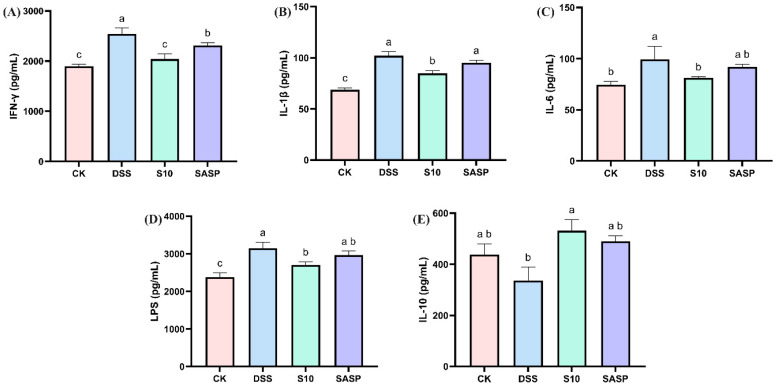
Effect of *Lactiplantibacillus plantarum* HYY-S10 on inflammatory factors in serum of mice with colitis. (**A**) IFN-γ; (**B**) IL-1β; (**C**) IL-6; (**D**) LPS; (**E**) IL-10. The letters a,b,c indicated significant difference between different groups (*p* < 0.05).

Comparative analysis revealed that DSS administration significantly upregulated pro-inflammatory mediators, including IFN-γ, IL-1β, IL-6, and LPS, while downregulating IL-10 compared to CK controls (*p* < 0.05), indicating both localized intestinal inflammation and systemic inflammatory responses. Notably, HYY-S10 intervention effectively reversed these inflammatory alterations, demonstrating significant reductions in IFN-γ, IL-1β, IL-6, and LPS levels, coupled with increase in IL-10 expression (*p* < 0.05, Figure 5A–E). This indicates that HYY-S10 may reduce inflammatory infiltration of the colon, primarily composed of neutrophils or macrophages, by decreasing production of pro-inflammatory factors.

### 3.6. HYY-S10 Improves Mouse Intestinal SCFAs

**Figure 6:**
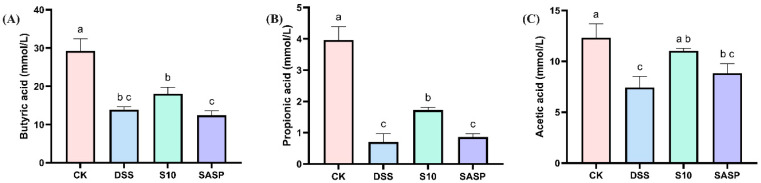
Effect of *Lactiplantibacillus plantarum* HYY-S10 on intestinal SCFAs in colitis mice. (**A**) Butyric acid; (**B**) Propionic acid; (**C**) Acetic acid. The letters a,b,c indicated significant difference between different groups (*p* < 0.05).

As shown in Figure 6, the levels of various SCFAs in the feces of DSS mice showed a decreasing trend, which may be related to the disruption of gut flora diversity by DSS. Compared with DSS-induced mice, HYY-S10 increased the levels of butyric acid (Figure 6A), propionic acid (Figure 6B) and acetic acid (Figure 6C) in mouse feces, suggesting that HYY-S10 was able to improve intestinal function by regulating the diversity of intestinal flora.

### 3.7. HYY-S10 Improves Intestinal Flora Composition in Mice

**Figure 7:**
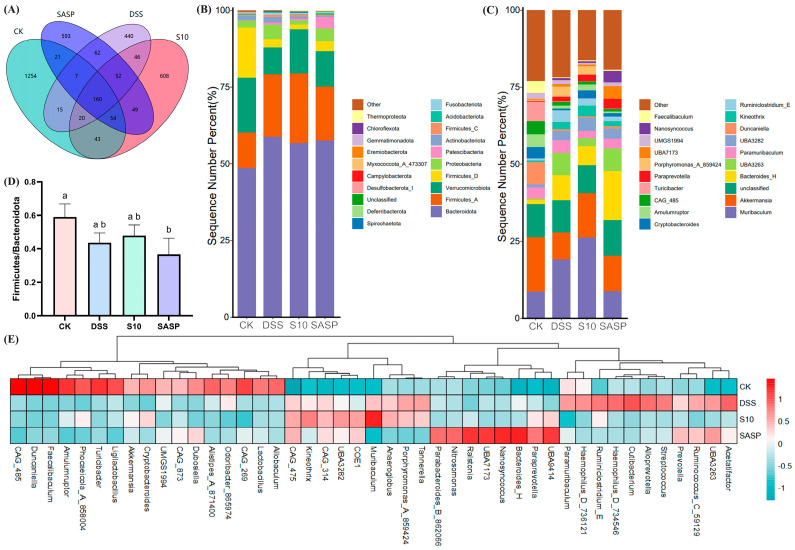
Effect of *Lactiplantibacillus plantarum* HYY-S10 on the intestinal flora of colitis mice. (**A**) Wayne’s plot; (**B**) Door level stacking map (%); (**C**) Genus horizontal stacking map (%); (**D**) F/B ratios; (**E**) Heat map of relative abundance (horizontal). The letters a,b indicated significant difference between different groups (*p* < 0.05).

16S rRNA sequencing analysis revealed distinct operational taxonomic unit (OTU) distributions among the experimental groups, as shown in the Venn diagrams (Figure 7A). The CK group showed the highest diversity with 1254 unique OTUs, followed by the S10 group (608 OTUs), SASP group (593 OTUs), and DSS group (440 OTUs). Intersection analysis revealed that the S10 group shared the highest number of OTUs (277) with the CK group, while the DSS group showed the least overlap (202 OTUs). A core microbiome of 160 OTUs was conserved across all groups. Comparative analysis revealed a significant reduction of 772 OTUs in the DSS group compared to the CK group. Notably, the HYY-S10 intervention resulted in significant microbial restoration, as evidenced by an increase of 230 OTUs compared to the DSS group. In addition, the results suggest that colitis leads to a decrease in the diversity of the gut flora in mice and that administration of HYY-S10 has the potential to increase the complexity of the gut microbiota.

As shown in Figure 7B, the four most predominant phyla at the phylum level were *Bacteroidota*, *Firmicutes*, *Verrucomicrobiota*, and *Proteobacteria*. The increased amounts of *Firmicutes* and *Proteobacteria* found in the gut of DSS mice are consistent with those found by Zhang et al. [18] and others. An increase in the abundance of proteobacteria is an indication of the proliferation of pathogenic bacteria in the gut [19]. The ratio of *Firmicutes* to *Bacteroidota* (F/B) serves as a key indicator of gut microbiota dysbiosis. As illustrated in Figure 7D, the F/B ratio in the DSS group depicted a decline; however, this ratio significantly improved (*p* < 0.05) following the intervention with HYY-S10. At the genus level (Figure 7C–E), our predominant groups were *Muribaculum*, *Akkermansia*, *Unclassified*, and *Bacteroidota*.

Comparative analysis of microbial composition revealed significant changes in the DSS-induced colitis group. Notably, the DSS group showed increased relative abundances of pro-inflammatory genera, including *Porphyromonas*, *Streptococcus*, and *Haemophilus*, when compared to the CK group. In contrast, there was a marked reduction in beneficial taxa, particularly *Akkermansia*, *Duncaniella*, and *Cryptobacteroide*. The depletion of *Akkermansia*, a genus strongly correlated with intestinal barrier integrity and gut homeostasis, as stated by Ghotaslou et al. [20], was particularly noteworthy as its reduced abundance is associated with impaired mucosal barrier function. Importantly, HYY-S10 administration effectively mitigated DSS-induced *Akkermansia* depletion, demonstrating its potential to restore gut microbial balance.

### 3.8. HYY-S10 Improves Intestinal Flora Diversity in Mice

**Figure 8:**
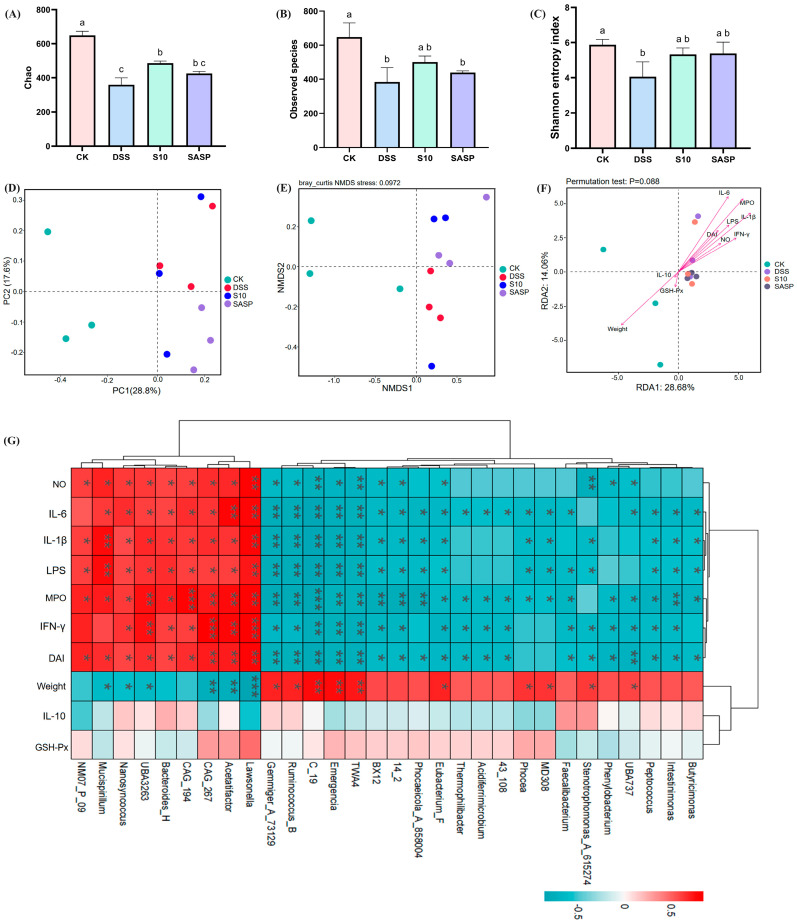
Effect of *Lactiplantibacillus plantarum* HYY-S10 on the diversity of intestinal flora in colitis mice. (**A**) Chao index; (**B**) Observed features index; (**C**) Shannon index; (**D**) PCoA; (**E**) NMDS; (**F**) Genus-level RDA analysis; (**G**) Genus-level Spearman correlation analysis. The letter abc indicated significant difference between different groups (*p* < 0.05). The symbols *, **, *** represent *p* < 0.05, 0.01 or 0.001, respectively.

Alpha diversity analyses demonstrate the diversity and richness of the gut microbial community. The Chao index (Figure 8A) and the Observed features index (Figure 8B) are frequently employed to analyze species abundance in samples, while the Shannon index measures microbial diversity (Figure 8C) [21]. Compared to the CK group, both community richness and diversity were significantly reduced (*p* < 0.05) in the DSS group; however, a notable recovery was observed (*p* < 0.05) in the S10 group following intervention. In Figure 8D, it is clear that all groups are distinctly separated and clustered into three separate categories. This observation indicates a significant difference in flora structure composition among these groups. Furthermore, non-metric multidimensional scaling (NMDS) analysis revealed a greater distance between the CK and DSS treatment groups compared to that between the S10 and DSS treatment groups. This finding suggests that colitis disrupts intestinal flora stability in mice, whereas HYY-S10 promotes restoration of intestinal flora structure in mice (Figure 8E).

### 3.9. Spearman Correlation Analysis

To gain a more in-depth understanding of the association between gut microbiota and colitis, we conducted a correlation analysis focusing on the top 30 genera. As illustrated in Figure 8G, this analysis used red to denote positive correlations and blue to represent negative correlations. This color-coded visualization facilitates clear discernment of the relationships between these genera and colitis. The levels of IL-1β, IL-6, IFN-γ, LPS, NO, MPO, and DAI showed positive correlated with *Lawsonella*, *Acetatifactor*, CAG_267, CAG_194, *Bacteroides*_H, UBA3263, and *Nanosyncoccus* (*p* < 0.05). In contrast, a negative correlation was observed (*p* < 0.05) with *Eubacterium*_F, 14_2, BX12, TWA4, *Emergencia*, C_19, *Ruminococcus*_B, *Gemmiger*_73129. Weight was positively correlated with UBA737, *Stenotrophomonas*_A_615274, MD308, *Phocea*, *Eubacterium*_F, TWA4, *Emergencia*, C_19, *Ruminococcus*_B, and *Gemmiger*_73129; and weight was negatively correlated with *Lawsonella*, *Acetatifactor*, UBA3263, *Nanosyncoccus*, and *Mucispirillum*. Among these, *Ruminococcus*_B is one of the most important gut microbiota genera for relieving colitis symptoms by fermenting dietary fiber to produce secondary metabolites (butyric acid and propionic acid) [22]. The correlation between IL-10, GSH-Px, and intestinal flora was not significant (*p* > 0.05). RDA analysis indicated a positive correlation between the levels of IFN-γ, IL-6, IL-1β, LPS, MPO, NO, and DAI with the intestinal flora present in the DSS, S10, and SASP groups (*p* < 0.05), arranged as follows: MPO > IL-6 > IL-1β > LPS > IFN-γ > DAI > NO. In the CK group, weight, IL-10, and GSH-Px showed a positive correlation with the intestinal flora, with weight displaying the strongest association (Figure 8F).

## 4. Discussion

UC represents a group of chronic gastrointestinal disorders characterized by recurrent inflammation, immune dysregulation, and alterations in gut microbiota composition and function [23]. Consequently, probiotics may offer an alternative or adjunct to conventional treatment. In this investigation, a murine colitis model was established through DSS administration to evaluate the therapeutic potential of HYY-S10 in intestinal inflammation. The results indicate that mice exposed to high concentrations of DSS exhibited clinical symptoms such as weight loss, colon atrophy, and bloody stools. Administration of DSS significantly compromised the structural integrity of the intestinal epithelial barrier in murine models, resulting in enhanced mucosal permeability. This pathological alteration was characterized by disrupted tight-junction complexes and compromised barrier function, as evidenced by increased translocation of macromolecules across the intestinal epithelium. Oxidative stress is closely associated with the progression of UC and serves as an indirect indicator of damage to the organism. MPO and NO levels in DSS-treated mice demonstrated significant neutrophil infiltration, which was further corroborated by histopathological examination via H&E staining. Dysregulated production of ROS has been implicated in the pathogenesis of UC. Excessive ROS accumulation disrupts intestinal barrier integrity, leading to increased permeability and amplification of the inflammatory cascade [24]. Notably, MPO, a marker of neutrophil activation, exacerbates oxidative tissue damage in inflamed regions, while the endogenous antioxidant GSH-Px counteracts this effect by scavenging ROS [25]. Furthermore, NO, generated from L-arginine by NOS, may contribute to mucosal injury under pathological conditions; its overproduction promotes ulcer formation via ROS-dependent mechanisms [26]. The consumption of HYY-S10 has been shown to mitigate this process significantly downregulating levels of oxidative mediators (such as MPO and NO), while simultaneously upregulating the levels of antioxidant mediators like GSH-Px. The amplification of oxidative stress coupled with enhanced neutrophil infiltration triggers a cascade of pro-inflammatory mediator release, culminating in the development of systemic inflammatory response syndrome with subsequent progression to multiple organ dysfunction syndrome in the intestinal microenvironment [27]. The aggravation of oxidative stress not only stimulates the release of IFN-γ from macrophages, but also induces the secretion of inflammatory factors such as IL-6 and IL-1β, ultimately leading to metabolic disorders in the intestinal mucosa. HYY-S10 increases the level of IL-10 in lamina propria cells, inhibits pro-inflammatory cytokines through negative feedback regulation, and modulates the differentiation and proliferation of macrophages, T cells, and B cells. This mechanism helps prevent excessive immune responses within the body [24].

Interactions between the intestinal flora and the body’s innate and adaptive immune systems are crucial for maintaining intestinal homeostasis and regulating inflammation [28]. Microbiota analysis revealed significant dysbiosis in DSS-induced colitis mice, demonstrating a marked microbial shift characterized by the enrichment of *Ruminicostridium*_E, *Paramuribaculum*, *Bacteroides*, and *Proteobacteria*, concurrent with depletion of *Verrucomicrobiota*, *Akkermansia*, and *Cryptobacteroides* compared to the CK groups. However, supplementation with HYY-S10 effectively alleviated these adverse changes while promoting the enrichment of beneficial microbiota. The therapeutic administration of HYY-S10 demonstrated remarkable efficacy in restoring microbial diversity indices within the gut ecosystem. Concurrently, this probiotic intervention significantly increased the proportional representation of beneficial bacterial taxa, in particular the genera *Firmicutes*, *Akkermansia*, *Muribaculum*, and *Ruminococcus*_B. As an endogenous mucin-degrading bacterium, *Akkermansia* has been shown to increase the rate of mucin utilization rate and mitigate LPS infiltration by reducing intestinal permeability, thereby alleviating the inflammatory response [29]. *Muribaculum* and *Ruminococcus*_B are capable of producing SCFAs from both endogenous and exogenous polysaccharides, which in turn promotes the growth and development of intestinal epithelial cells while decreasing macrophage infiltration of inflammatory factors [30]. SCFAs, the primary microbial metabolites generated through the anaerobic fermentation of dietary fibers, play a crucial role in regulating intestinal homeostasis. Functionally, these microbial-derived metabolites not only promote the expansion of commensal microbiota by creating an acidic luminal environment, but also constitute the predominant energetic substrates for colonocytes [31]. Importantly, recent research has elucidated the pleiotropic immunoregulatory capacities of SCFAs, highlighting their promising therapeutic applications in the management of inflammatory pathologies. Mechanistically, butyrate, functioning as a competitive inhibitor of histone deacetylases, effectively attenuates LPS-triggered macrophage polarization, consequently downregulating the production of proinflammatory cytokines, including NO and IL-6. In parallel, acetate and propionate exhibit comparable immunomodulatory effects through distinct pathways [32]. The altered gut microbiota composition in colitis-induced murine models significantly impairs SCFAs biosynthesis and metabolic homeostasis. Oral administration of HYY-S10 effectively modulates this dysbiosis, leading to substantial elevation of key SCFAs, including acetate, propionate, and butyrate, within the intestinal lumen.

## 5. Conclusions

In summary, our findings demonstrate that *Lactiplantibacillus plantarum* HYY-S10 exerts protective effects against DSS-induced colitis through dual mechanisms of immune regulation and microbial modulation. These results provide compelling evidence for the potential application of HYY-S10 as a novel probiotic-based therapeutic intervention in UC management.

## Figures and Tables

**Table 1:** DAI Scoring Rules.

Score	Percentage of Weight Loss	Fecal Viscosity	Fecal Occult Blood
0	0	Normal	Negative
1	1–5%	Soft stool	Pale blue
2	5–10%	Mucoid stools	Blue
3	10–20%	Liquid stools	Dark blue
4	>20%	—	Bloody stools

## Data Availability

The datasets presented in this article are not readily available because the data are part of an ongoing study. Requests to access the datasets should be directed to Yanyan Huang.
